# Transcriptome analysis reveals key genes involved in the regulation of nicotine biosynthesis at early time points after topping in tobacco (*Nicotiana tabacum* L*.*)

**DOI:** 10.1186/s12870-020-2241-9

**Published:** 2020-01-20

**Authors:** Yan Qin, Shenglong Bai, Wenzheng Li, Ting Sun, David W. Galbraith, Zefeng Yang, Yun Zhou, Guiling Sun, Bingwu Wang

**Affiliations:** 1 Key Laboratory of Plant Stress Biology, State Key Laboratory of Crop Stress Adaptation and Improvement, State Key Laboratory of Cotton Biology, School of Life Sciences, Henan University, Kaifeng, 475004 China; 2 Tobacco Breeding Center, Yunnan Academy of Tobacco Agricultural Sciences, Kunming, 650021 Yunnan China; 3 School of Plant Sciences and Bio5 Institute, The University of Arizona, Tucson, AZ 85721 USA; 4 Jiangsu Key Laboratory of Crop Genetics and Physiology/Key Laboratory of Plant Functional Genomics of the Ministry of Education/Jiangsu Key Laboratory of Crop Genomics and Molecular Breeding, Agricultural College of Yangzhou University, Yangzhou, 225009 China

**Keywords:** Tobacco, Topping, Transcriptome analysis, Differentially expressed genes, Nicotine biosynthesis and regulation

## Abstract

**Background:**

*Nicotiana tabacum* is an important economic crop. Topping, a common agricultural practice employed with flue-cured tobacco, is designed to increase leaf nicotine contents by increasing nicotine biosynthesis in roots. Many genes are found to be differentially expressed in response to topping, particularly genes involved in nicotine biosynthesis, but comprehensive analyses of early transcriptional responses induced by topping are not yet available. To develop a detailed understanding of the mechanisms regulating nicotine biosynthesis after topping, we have sequenced the transcriptomes of *Nicotiana tabacum* roots at seven time points following topping.

**Results:**

Differential expression analysis revealed that 4830 genes responded to topping across all time points. Amongst these, nine gene families involved in nicotine biosynthesis and two gene families involved in nicotine transport showed significant changes during the immediate 24 h period following topping. No obvious preference to the parental species was detected in the differentially expressed genes (DEGs). Significant changes in transcript levels of nine genes involved in nicotine biosynthesis and phytohormone signal transduction were validated by qRT-PCR assays. 549 genes encoding transcription factors (TFs), found to exhibit significant changes in gene expression after topping, formed 15 clusters based on similarities of their transcript level time-course profiles. 336 DEGs involved in phytohormone signal transduction, including genes functionally related to the phytohormones jasmonic acid, abscisic acid, auxin, ethylene, and gibberellin, were identified at the earliest time point after topping.

**Conclusions:**

Our research provides the first detailed analysis of the early transcriptional responses to topping in *N. tabacum*, and identifies excellent candidates for further detailed studies concerning the regulation of nicotine biosynthesis in tobacco roots.

## Background

For tobacco (*Nicotiana tabacum* L.) plants, topping (defined as the removal of the flowering head and young leaves) is an essential cultivation practice. Topping switches the plant from a seed reproductive to a leaf vegetative phase, and this significantly increases leaf nicotine contents [1]. A number of studies, employing a variety of experimental techniques, have investigated tobacco responses to topping [2–4]. An up-regulation of nicotine biosynthesis, found to occur exclusively in roots, and particularly in growing root tips, is one of the typical responses of tobacco plants to topping [5].

Nicotine plays pivotal roles both in establishing the commercial quality of tobacco, and in defending plants against herbivores. Biosynthesis of nicotine, a secondary metabolite associated with the tobacco stress response, is reproducibly promoted by topping in tobacco roots [6]. Nicotine comprises two main nitrogen-containing rings, the pyrrolidine ring and the pyridine ring [7]. Biosynthesis of the pyrrolidine ring involves arginine decarboxylase (ADC) [8], ornithine decarboxylase (ODC) [9], S-adenosylmethionine decarboxylase (SAMDC), S-adenosyl-L-methionine synthetase (SAMS), putrescine N-methyltransferase (PMT) [10, 11], and N-methylputrescine oxidase (MPO) [12, 13]. Biosynthesis of the pyridine ring starts with the nicotinic acid dinucleotide (NAD) biosynthetic pathway. Enzymes participating in the early metabolic conversion steps of this pathway include aspartate oxidase (AO), quinolinate synthase (QS), and quinolinic acid phosphoribosyl transferase (QPT) [14–16]. The A622 gene (encoding an isoflavone reductase-like protein) is responsible for nicotine ring coupling, and the BBL genes (encoding berberine bridge enzyme-like proteins) are involved in the subsequent oxidation step that leads to nicotine [17, 18].

A recent report [19] employed suppression subtractive hybridization (SSH) techniques to further examine the transcriptional responses of tobacco roots during the first 24 h after topping. Of the 129 high quality expressed sequence tags identified as representing DEGs, most were involved in stress/defense, in secondary metabolism, and in signaling/transcription [19]. The regulation of nicotine biosynthesis has long been considered a complex physiological response, and many TFs are directly or indirectly involved in its regulation [20, 21]. Further insights into the transcriptional regulation of the nicotine biosynthetic pathway have come from the analysis of two subtractive cDNA libraries of jasmonate-treated *Nicotiana benthamiana* roots, and through examination of the effects of virus-induced gene silencing (VIGS) technologies. Of the sixty-nine TFs, six (from three TF families) affect nicotine metabolism, with *NbbHLH1* and *NbbHLH2* (basic helix-loop-helix) genes positively regulating the jasmonate activation of nicotine biosynthesis, as evidenced by overexpression [22].

Although specific genes regulating nicotine synthesis after tobacco topping have been identified, a detailed description of the transcriptional regulatory network that responds to topping is not available. The situation is further complicated by the allotetrapoid status of *N. tabacum*, formed through the hybridization of *N. sylvestris* (S-subgenome) and *N. tomentosiformis* (T-subgenome), and how these two subgenomes respond to topping is unclear. In this study, we have sequenced tobacco root transcriptomes at seven different time points (0, 0.5, 1, 3, 5, 8 and 24 h) after topping. These time points were chosen to identify candidate genes associated with the regulation of nicotine biosynthesis at the earliest stages, as well as to allow discovery of upstream regulators of nicotine synthesis through clustering of the time-course profiles of TF gene expression, and to compare the responses of the two subgenomes to topping. This comprehensive approach to characterization of the transcriptional responses of tobacco, especially focusing on the early regulation of nicotine biosynthesis, should serve to advance genetic improvement in this crop.

## Results

### Transcriptome sequencing and quality assessment

Total RNA of tobacco roots, isolated separately from 18 individual plants, was employed for RNA sequencing (RNA-Seq) library construction. The 18 RNA-Seq libraries were sequenced using the Illumina platform. After filtering out low quality sequences (quality scores < 25), 105 Gb of cleaned data was obtained, representing approximately 6 Gb per sample. The cleaned sequence GC content varied from 42.1 to 42.7% (Additional file 6: Table S1). The mapping rates for the cleaned sample reads aligned against the reference genome sequence ranged from 91.6 to 97.8% (Additional file 6: Table S1). The sequencing quality and gene expression levels were generally consistent across the sequenced samples (Additional file 1: Figure S1).

### Identification and verification of differentially expressed genes (DEGs)

**Fig. 1:**
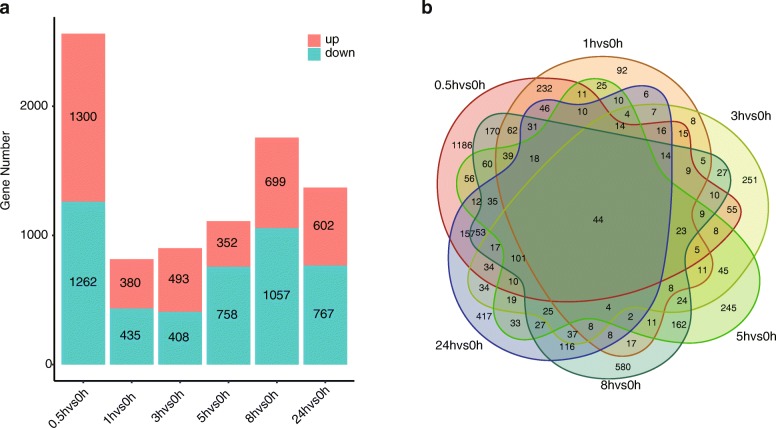
DEGs statistics at different stages after tobacco topping. **a** In the stacked bar-graphs, the up-regulated DEGs are located in the red regions, and the down-regulated DEGs in the blue regions. **b** Venn diagram of DEGs at different time points after topping in tobacco

The expression levels of the genes from the tobacco transcriptomes were calculated and normalized to FPKM values (Fragments Per Kilobase of transcript per Million fragments mapped). The values of Pearson’s Correlation Coefficient across biology replicates exceeded 0.82. In terms of the correlation between samples from different time points, some samples displayed higher values with those from other time points. For example, BWR3-2A showed a correlation coefficient of 0.96 as compared to BWR24-1A, and 0.95 with BWR5-2A (Additional file 2: Figure S2). Further experiments will be required to elucidate this unexpected observation. Through comparison of the samples at each time point to the t = 0 sample, and using a fold-change (FC) > 2, and a false discovery rate (FDR) < 0.05 as selection criteria, 4830 DEGs were identified after topping. An almost identical number (2082 and 2075 genes) came from the *N. tomentosiformis* and *N. sylvestris* genomes, respectively (Additional file 7: Table S2). Notably, the number of DEGs at 0.5 h (2,562) was much greater than those at any other time point, indicating that more genes respond to topping at earlier times. The number of DEGs dropped to its lowest level (815) at 1 h after topping (Fig. 1a). However, a second burst of differential gene expression was observed at t = 8 h (1,756), followed by a decrease at t = 24 h (Fig. 1a). The results imply that the *N. tabacum* root produces two discrete peaks of transcriptional activity, at 0.5 h and 8 h after topping. This result is consistent with the gene numbers identified as specifically induced at each of the six time points after topping, the largest number being 1186 at t = 0.5 h and the second highest number being 585 at t = 8 h after topping (Fig. 1b).

**Fig. 2:**
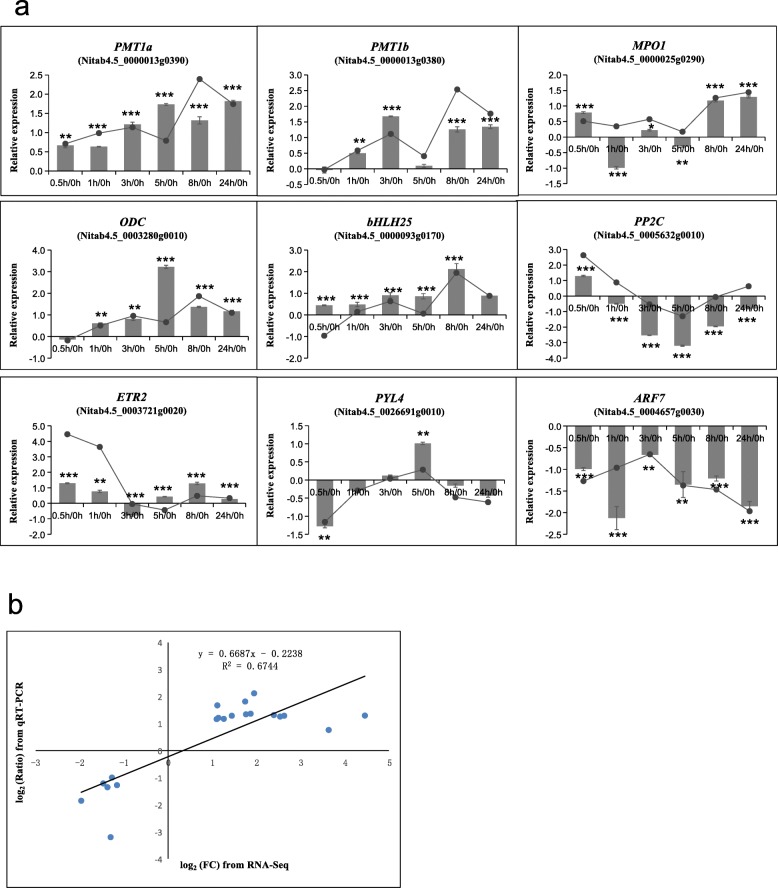
Validation of RNA-Seq data by qRT-PCR. **a** Expression levels of 9 randomly-selected DEGs of the nicotine anabolic pathway as measured by qRT-PCR (the columns) and the corresponding expression trends measured by RNA-Seq (the lines). The error bars represent SDs (*n* = 3). Asterisks represent significantly different transcript levels between the topping treatment and control plants at the indicated times. (t-test; *, *P* < 0.05; **, *P* < 0.01; ***, *P* < 0.001). **b** Correlation analysis of fold-change data between qRT-PCR and RNA-Seq. Scatterplots are generated from the log_2_ expression ratios of qRT-PCR analyses (*x*-axis) and from RNA-Seq analyses (*y*-axis). Each scatter point depicts a time point at which significant differences in gene expression levels were found. The equation of the linear regression relationship and the associated correlation coefficient (R^2^) are provided

To validate the transcriptional results obtained by RNA-Seq, we selected nine genes related to nicotine biosynthesis and phytohormone signal transduction, and examined their transcriptional responses by qRT-PCR. The expression trends of these genes analyzed by qRT-PCR were consistent with the RNA-Seq analysis performed at the corresponding time points (Fig. 2). The changes of selected DEGs obtained by RNA-Seq analysis had good correlations with those obtained by qRT-PCR (R^2^ = 0.674). These results confirm that the alterations in gene expression detected by RNA-Seq accurately reflect transcript differences at the different time points after topping.

### Functional classification and enrichment analysis of DEGs

4830 DEGs showing significant variation at the different time points after topping were selected for further analysis. Based on their relative expression levels, the DEGs were divided into different categories using hierarchical clustering, being distinguishable in terms of the temporal patterns of the transcriptional responses of the roots at the various time points after topping (Additional file 3: Figure S3). The predicted functions of the DEGs were then obtained from their GO (Gene Ontology) annotations, and using KEGG (Kyoto Encyclopedia of Genes and Genomes) pathway analysis. According to GO term annotation, the DEGs were distributed across 42 functional terms, as follows: 19 terms for biological process, 12 terms for molecular functions, and 11 terms for cellular component (Additional file 4: Figure S4).

**Fig. 3:**
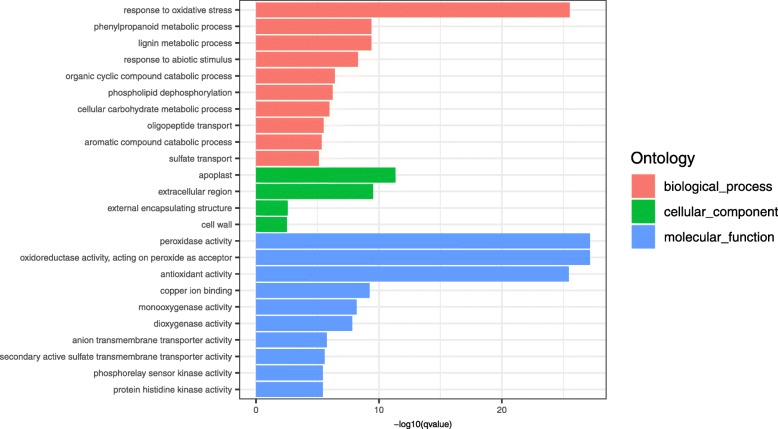
Gene Ontology (GO) term enrichment analysis. Significantly enriched GO terms were selected based on a FDR < 0.05. GO terms of the categories of Biological Processes, Cellular Components, and Molecular Functions are depicted in red, green, and blue, respectively

GO enrichment analyses were performed to classify the putative functions of DEGs in the comparisons of libraries prepared from the different time points (Fig. 3). The DEGs in GO enriched categories of biological process were mainly involved in response to oxidative stress (GO:0006979), the phenylpropanoid metabolic process (GO:0009698), the lignin metabolic process (GO:0009808), and response to abiotic stimulus (GO:0009628). The DEGs of GO enriched categories of cellular component were mainly involved in the apoplast (GO:0048046), the extracellular region (GO:0005576), the external encapsulating structure (GO:0030312), and the cell wall (GO:0005618). The DEGs of GO enriched categories of molecular function were mainly associated with peroxidase activity (GO:0004601), antioxidant activity (GO:0016209), and a series of transporter activities (GO: GO:0006857, GO:0008272, GO:0008509, and GO:0008271) (Fig. 3).

**Fig. 4:**
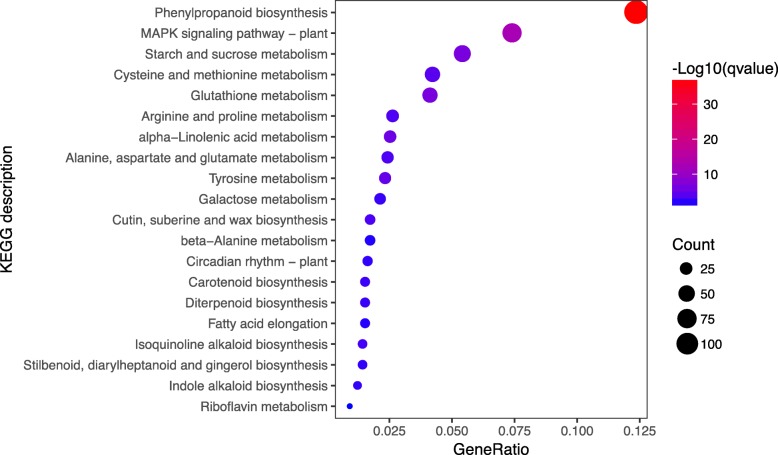
KEGG enrichment analysis. Each circle in the figure represents a KEGG metabolic pathway, and the number of genes enriched in a pathway corresponds to the size of the circle. The degree of significance of the enrichment of DEGs in a pathway is represented by -log_10_ (qvalue). The abscissa indicates the ratio of the number of DEGs annotated to a particular pathway to the number of the DEGs annotated to all pathways

To further investigate the functions of differentially expressed transcripts in response to topping, we performed enrichment analyses by mapping the sequences to the KEGG database categories. The DEGs with KEGG annotation were assigned to 28 classes, mainly related to signal transduction (221), carbohydrate metabolism (212), biosynthesis of other secondary metabolites (166), and metabolism of terpenoids and polyketides (69) (Additional file 5: Figure S5). KEGG enrichment analyses also indicated the DEGs were significantly enriched in the main pathways of phenylpropanoid biosynthesis (ko00940), of starch and sucrose metabolism (ko00500), and in the plant MAPK signaling pathway (ko04016, their responses to wounding and their roles in the biosynthesis of secondary metabolism have been illustrated previously [23–25] (Fig. 4).

### DEGs involved in nicotine synthesis and transport

**Fig. 5:**
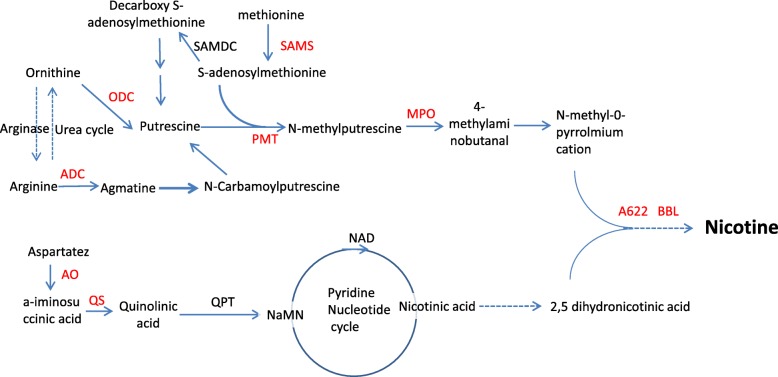
The DEGs involved in nicotine biosynthesis. Solid and dashed lines indicate defined and undefined reactions, respectively, with the DEGs shown in red. Abbreviations: ADC, arginine decarboxylase; ODC, ornithine decarboxylase; SAMS, S-adenosyl-L-methionine synthetase; SAMDC, S-adenosylmethionine decarboxylase; PMT, putrescine N-methyltransferase; MPO, N-methylputrescine oxidase; AO, aspartate oxidase; QS, quinolinate, synthase; QPT, quinolinic acid phosphoribosyl transferase; A622, isoflavone reductase-like protein; BBLs, berberine bridge enzyme-like proteins. The genes with significantly up-regulated transcription levels are shown in red

We further investigated whether the genes activated by topping were involved in nicotine biosynthesis and transport. As expected, nine gene families involved in nicotine biosynthesis (*AO*, *QS*, *ODC*, *ADC*, *SAMS*, *PMT*, *A622, MPO*, and *BBL*) (Fig. 5), and two gene families involved in nicotine transport (*MATE*, *NUP*), as identified by showing at least 93% identities with the primary sequences of previously reported enzymes, were found in the DEG dataset (Additional file 8: Table S3). All genes showed transcriptional up-regulation, with most being up-regulated at 8 h and 24 h after topping; our qPCR assay also verified the expression changes of four genes (*PMT1a, PMT1b, MPO, ODC*) at the corresponding time points (Fig. 2). Both gene families encoding MATE and NUP in nicotine transport were found to be up-regulated (Additional file 8: Table S3). Similar to the situation across all DEGs, most of those involved in nicotine synthesis and transport were found in both subgenomes. One DEG encoding AO was derived from the T-subgenome and all the DEGs encoding MPO and ODC were from the S-subgenome.

### Transcription factors (TFs) of DEGs, and gene clustering by expression patterns

**Fig. 6:**
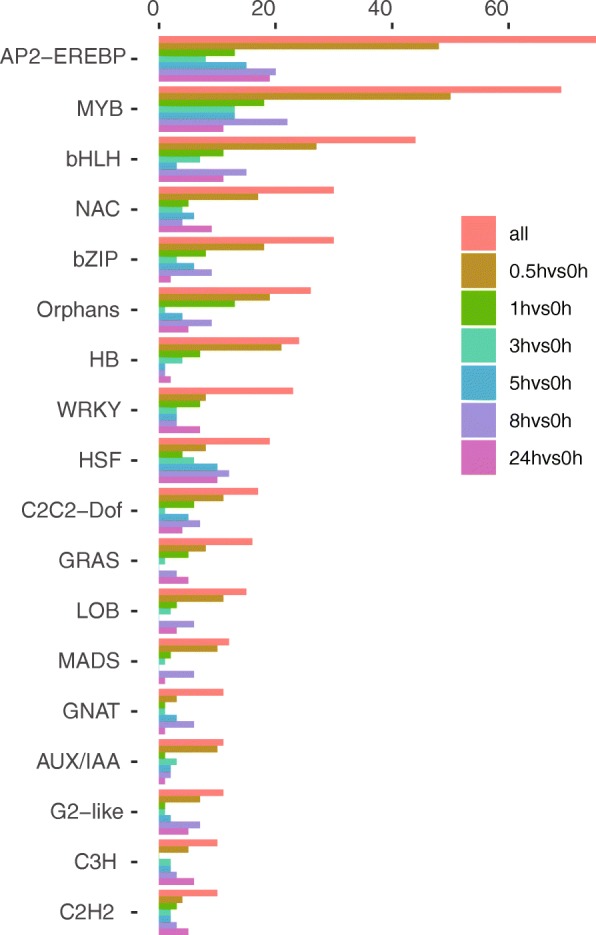
TFs classification of DEGs in tobacco. DEGs at different time points after topping are represented by different colors, the abscissa representing the number of transcription factors at each time point

To investigate the upstream regulatory mechanisms of nicotine biosynthesis after topping, we next focused on the types of TFs represented in the DEGs from the tobacco root transcriptome. In our study, a total of 549 DEGs encoding TFs were identified (Additional file 9: Table S4), being divided into 49 TF families. Amongst these, the number of TFs was highest at t = 0.5 h (355), accounting for 65% of all TFs, with 240 being up-regulated and 115 down-regulated. This was followed by the t = 8 h timepoint (253), accounting for 46% of all TFs, with 94 being up-regulated and 159 down-regulated. This suggests that many TFs genes participate in instant early gene activation. Notably, and representing most of these TFs, 18 families were found to contain more than 10 gene members: AP2-EREBP (75), MYB (69), bHLH (44), NAC (30), bZIP (30), Orphans (26), HB (24), WRKY (23), HSF (19), C2C2-Dof (17), GRAS (16), LOB (15), MADS (12), GNAT (11),AUX / IAA (11), G2-like (11), C3H (10) and C2H2 (10) (Fig. 6).

**Fig. 7:**
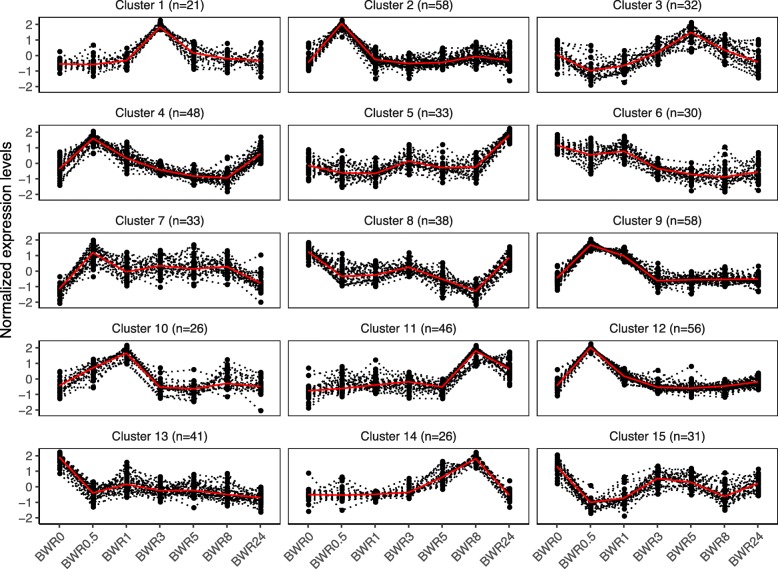
The clustering of gene expression pattern of DEGs on the TFs and the genes involved in nicotine biosynthesis and transport across different time points after topping in tobacco. The x-axis represents treatment conditions and the y-axis represents centralized and normalized expression values. The red lines indicate the mean expression trends of the TFs (dotted lines) belonging to each cluster. The gene number is marked following the cluster ID

To further examine the contributions of specific TFs to the regulatory network of nicotine biosynthesis, we performed clustering using the 549 TFs and the up-regulated structural genes associated with the nicotine biosynthesis pathway. Fifteen clusters showing similar expression profiles were obtained (Fig. 7 and Additional file 10: Table S5). It can be observed that several clusters are similar but with minor differences. For instance, the TFs of Clusters 2 and 12 were up-regulated at t = 0.5 h, and the TFs in Cluster 9 and 10 were up-regulated at 0.5–1 h. They then returned to the expression levels found before topping (Fig. 7). Notably, most of the up-regulated DEGs in nicotine biosynthesis were in Cluster 11 (20 DEGs), which displayed the greatest up-regulation at t = 8 h and at t = 24 h after topping (Fig. 7 and Additional file 10: Table S5). Seventeen genes from the bHLH family and the AP2-EREBP family were found in Cluster 11, including ERF189 (Nitab4.5_0003090g0030 and Nitab4.5_0015055g0010), and ERF91 (Nitab4.5_0004620g0030) (Additional file 10: Table S5).

### DEGs connected with phytohormone signal transduction

As phytohormones are known to quickly respond to tobacco topping and to also influence nicotine biosynthesis in tobacco roots, we examined the role of phytohormone signal transduction in the transcriptional responses induced by topping. We identified 336 DEGs, including those related to the biosynthesis, metabolism and action of auxin (IAA), abscisic acid (ABA), ethylene, gibberellin (GA), and jasmonic acid (JA) (Additional file 11: Table S6). The 53 DEGs involved in IAA signal transduction included the ARF (auxin response factor) family (4), the AUX/IAA (auxin responsive protein) family (11), the AUX1 (amino acid transporter protein) family (22), the GH3 (GH3 auxin-responsive promoter) family (7), and the SAUR (auxin responsive SAUR protein) family (9). Most of the DEGs associated with the IAA signaling pathway showed significant up-regulated expression changes, 21 of 36 genes being up-regulated at t = 0.5 h, and 10 of 18 genes being up-regulated at t = 8 h. For the ABA signal transduction pathway, six gene families were identified, including the PYL/PYR (abscisic acid receptor) family (5), the SAPK (Serine threonine protein kinase) family (2), the PP2C (protein phosphatase 2C) family (28), the CIPK (CBL-interacting protein kinase) family (19), the CDPK (Calcium-dependent protein kinase) family (9), and the Calmodulin (Calmodulin-like protein) family (8). 43 expression changes were detected at t = 0.5 h, and 19 at t = 8 h. 83 DEGs were implicated in ethylene signaling, including the AP2-EREBP (ethylene responsive transcription factor) family (75), and the ETR (ethylene receptor) family (8), with most DEGs identified at t = 0.5 h (55). The GA and the JA signaling pathways (four and three gene families, respectively) also showed significant transcriptional changes after topping.

### Quantification of phytohormones and nicotine

**Fig. 8:**
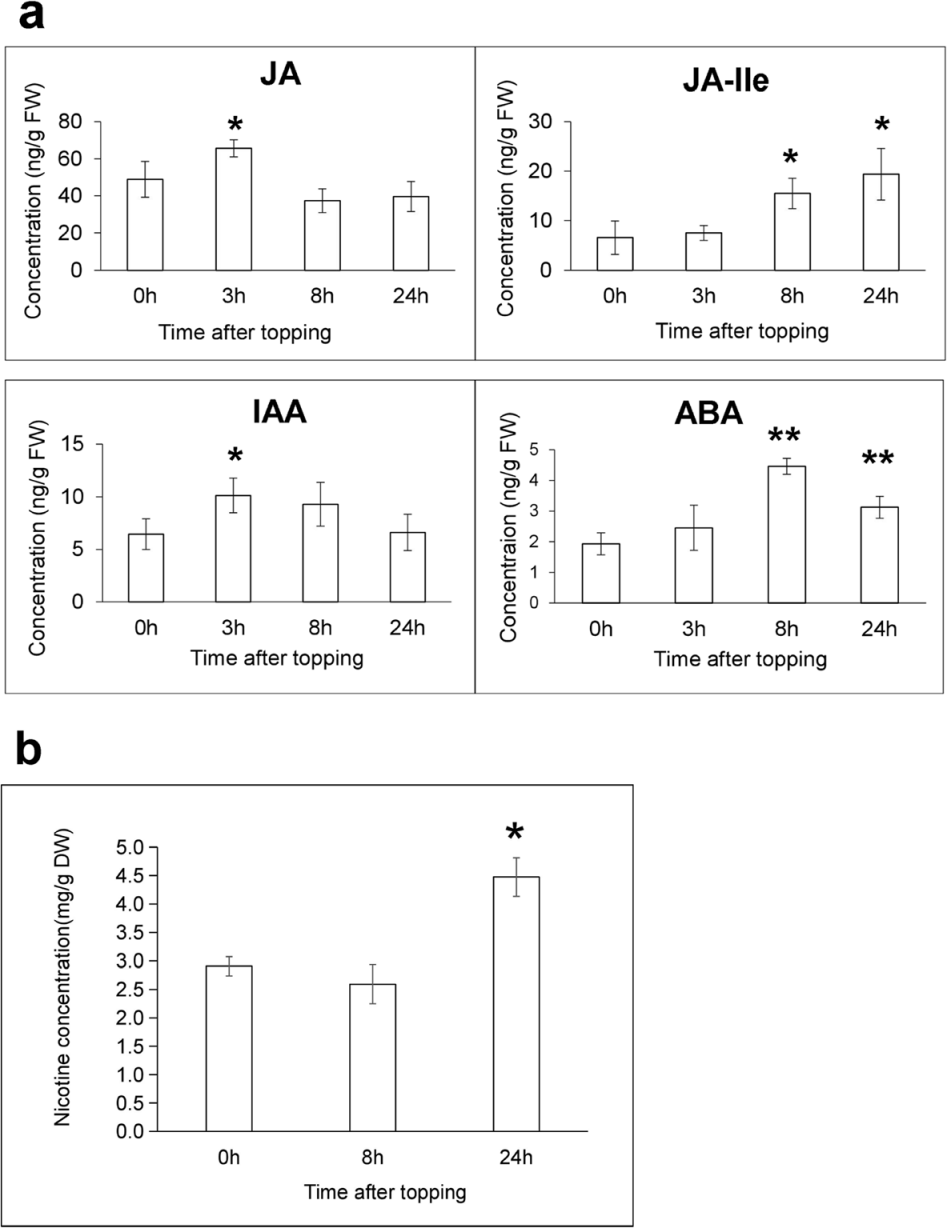
Mean (+SE) concentrations of phytohormones and nicotine from three replicates of roots harvested after topping treatment within the indicated times. **a** Mean (+SE) concentrations of JA, JA-Ile, IAA, and ABA from *N. tabacum* roots were measured using HPLC-MS/MS within 24 h after topping. **b** The level of nicotine from roots within 24 h after topping treatment, untreated plants served as controls. Asterisks represent significantly different hormone and nicotine levels between control and treatment plants after the indicated times. (t-test; *, *P* < 0.05; **, *P* < 0.01)

Phytohormones play a vital role in regulating plant defense and development. To gain insights into the mechanisms whereby phytohormones affect the responses of tobacco to topping, we measured the levels of IAA, JA, JA-Ile, and ABA in the root samples at the various timepoints after topping. Both JA and auxin signaling pathways were induced by topping at t = 3 h. JA levels at t = 3 h were significantly increased by almost 34% (*P* = 0.035, paired *t* test), and reduced by 23.5 and 18.9% at t = 8 h and t = 24 h (Fig. 8). The dynamics of JA-Ile levels elicited by topping closely followed those of JA, the levels of JA-Ile significantly increasing to about 3-fold at t = 24 h (*P* = 0.014, paired *t* test) as compared with untreated plants. The levels of IAA significantly increased at t = 3 h (*P* = 0.024, paired *t* test), whilst declining to initial levels at t = 24 h (Fig. 8). The levels of ABA gradually increased to 2.3-fold at t = 8 h (*P* = 0.0003, paired *t* test), and to 1.6-fold at t = 24 h (*P* = 0.009, paired *t* test), as compared to the untreated plants. We also measured nicotine levels after topping. Our analyses indicated the nicotine levels significantly increased to 1.5-fold at t = 24 h (*P* = 0.01, paired *t* test) after topping (Fig. 8).

## Discussion

### Transcriptome sequencing and DEGs responses to topping

Nicotine is a characteristic secondary product of tobacco. In most *Nicotiana* species, it is synthesized in the roots, being then transported to the leaves where it accumulates [26]. Nicotine synthesis and accumulation is controlled not only by various environmental cues but also by managerial practices including topping [2, 6]. The factors controlling the topping-induced increase in alkaloid biosynthesis are not well understood, but involve a complex physiological response in the plant as a result of altered phytohormone induced signaling [11]. In order to better understand the mechanism of the tobacco response to topping, we have sequenced and analyzed the transcriptomes of *N. tabacum* roots at early time points after topping. We identified a total of 4830 topping-responsive DEGs, with representatives being distributed across a number of different molecular functional categories, including secondary metabolism, plant hormone signaling transduction, stress defense, and other metabolism.

### DEGs involved in nicotine biosynthesis and subgenome transcriptional preference

We detected 1.5-fold changes in nicotine levels at t = 24 h after topping with each biological replicates containing 4 individual plants, which is consistent with previous report [27]. It was worth to mention that no significant changes of nicotine content were detected at this time point with each biological replicates containing a single plant (data not shown), indicating that the individual plants response differently in the short time of decapitation. We then inspected more closely the transcriptional changes of genes known to be involved in nicotine biosynthesis and transport. Nine gene families in the nicotine biosynthesis pathway were identified within the DEGs. Their functions included pyridine ring synthesis (*AO* and *QS*), pyrrolidine ring synthesis (*ODC*, *ADC*, *PMT*, *SAMS*, *MPO*), and the coupling of the two nicotine rings (*A622* and *BBL*). Ornithine decarboxylase (ODC) catalyzes the first and rate-limiting step of polyamine biosynthesis that converts ornithine into putrescine. Down-regulation of *ODC* transcript levels using RNAi led to lower leaf levels of nicotine in *N. tabacum* [28, 29]. Correspondingly, in our study, one *ODC* gene was significantly up-regulated at t = 8 h and t = 12 h. A second example involves PMT, which converts putrescine into N-methylputrescine [30]. In that previous study, five *PMT* genes were investigated (*NtPMT1a*, *NtPMT1b*, *NtPMT2*, *NtPMT3* and *NtPMT4*). Transcripts derived from *NtPMT2* and *NtPMT1b* showed the greatest increase in abundance (about 3-fold) during the first 24 h after topping [31]. In line with these findings, the expression levels of all the five PMT genes in our study were significantly up-regulated at different time points after topping (Additional file 7: Table S2). A further example is provided by SAMS, which indirectly contributes to nicotine biosynthesis by supplying the S-adenosylmethionine cofactor for the PMT reaction [32]. In our study, five *SAMS* genes were significantly up-regulated at early time points after topping (Additional file 8: Table S3).

The enzyme QPT plays a critical role in the synthesis of the pyridine moiety of nicotine in *Nicotiana*, in addition to its ubiquitous role in NAD(P)(H) synthesis [33]. The tobacco genome contains two duplicated *QPT* genes (designated *QPT1* and *QPT2*). *QPT1* is expressed at a constitutive basal level in all plant tissues, with somewhat higher levels of expression within the apical meristem. In comparison, *QPT2* is expressed exclusively in the tobacco root and is regulated coordinately with other structural genes for nicotine biosynthesis [33, 34]. Although *QPT1* and *QPT2* were not present in our list of DEGs, the gene of QPT2 (Nitab4.5_0000742g0010) showed significant up-regulation at 3 h and 24 h in our qPCR assay (data not shown).

Finally, we consider *A622*, which is expressed in the root, and may be involved in the final condensation reaction of nicotine biosynthesis [35]. The capacity of *N. glauca* to produce anabasine was markedly reduced when an RNAi approach was used to down-regulate gene expression, thereby decreasing A622 protein levels. This resulted in plants having almost undetectable levels of pyridine alkaloids in their leaves, even after suffering damage to apical tissues [18]. In our work, the expression of *A622* was significantly up-regulated at t = 8 h and t = 24 h after topping (Additional file 7: Table S2), implying that *A622* positively regulates the biosynthesis of nicotine in the final coupling ring step. Consistent with the results for *A622*, *BBL* genes were also found to be induced after topping, as previously reported [17].

*N. tabacum* is hypothesized to be a consequence of hybridization of two parental genomes (*N. sylvestris* and *N. tomentosiformis*). *N. tomentosiformis* displayed much lower nicotine levels than *N. sylvestris* in both roots and leaves [36]. All DEGs, especially those involved in nicotine biosynthesis and transport, showed no obvious preference to any of the two subgenomes. More experimental work will be required to elucidate the molecular basis of heterosis and the dramatic domestication selection following the hybridization.

### Differential expressed transcription factors related to regulation of nicotine synthesis

The pattern of expression changes induced by topping for the structural genes of nicotine biosynthesis was initially derived from bioinformatics analyses of sequence data, but was confirmed by the qRT-PCR experiments. It was therefore reasonable to speculate that the sequence data could also be used to identify the TF(s) that actively regulate the production of nicotine at the early time points after topping. As reported previously, many TFs play important roles in regulating nicotine biosynthesis, including members of the AP2/EREBP, bHLH, ARF, and WRKY families [37, 38]. The AP2/EREBP family is the largest TF family in the tobacco genome [39], and ERF-type TFs of the group IX subfamily, including *ERF1, ERF189*, and *ERF32*, have been recently identified as direct regulators of the structural genes of nicotine biosynthesis [22, 33, 40]. The second largest class of TFs shown to induce alkaloid biosynthesis in *Nicotiana* is the MYC2-like bHLH family. *MYC2*, belonging to the bHLH-family of TFs, is a key component in conserved jasmonate signaling [41]. It positively regulates nicotine production either directly, through G-box-mediated binding and activation of nicotine structural genes, or indirectly, through the activation of the *ERF* genes [42, 43]. Wang et al. [44] found that overexpression of *NtMYC2a* led to great enhancement, under field testing, of nicotine levels in the transgenic lines. Although the mechanism by which *ARF1* regulates nicotine synthesis remains unclear, VIGS (virus-induced gene silencing) of *bHLH3* and *ARF1* results in a significant increase in nicotine content as compared to control plants [22]. In addition, *WRKY-R1*, the Group II member of the WRKY family, was specifically and highly expressed in tobacco roots. This suggests it regulates the expression of genes related to nicotine synthesis, like *PMT* [37].

Screening the DEGs in our study led to the identification of 549 DEGs annotated as TFs, including members of the *AP2/EREBP* (75), *bHLH* (44), *WRKY* (23), and *ARF* (4) families. (Fig. 6 and Additional file 9: Table S4). To elucidate patterns of co-regulation of TFs, we clustered all DEG TFs along with the structural genes involved in nicotine biosynthesis. We found that Cluster 11 contained 20 of 28 structure and transporter genes in the DEGs, as well as a total of 17 genes from the bHLH and AP2-EREBP families. We selected one of seven genes in the bHLH family (Nitab4.5_0000093g0110) for study using RNAi, finding the resultant plant showed an altered nicotine level (unpublished data), and implying an important regulatory role in nicotine biosynthesis. Other newly identified *AP2/EREBP, bHLH, WRKY, ARF, MYB*, and *NAC* TF genes might also be involved in nicotine biosynthesis, since all of these TF families have been described as functioning in the regulation of plant secondary metabolism [45–47]. These are therefore good targets for further experiments.

### Involvement of DEGs responsive to topping in phytohormone signal transduction

Plant hormones play pivotal roles in regulating numerous aspects of plant growth and development, including secondary metabolism. Five major classes of plant hormones are known to be involved in the regulation of nicotine biosynthesis, including JA, IAA, GA, ethylene, and ABA [32, 48–51]. It is known that JA treatment effectively induces nicotine biosynthesis in tobacco by regulating genes encoding enzymes of nicotine biosynthesis, including *QPT*, *ODC*, and *PMT* [34, 38, 52, 53]. Yang et al. [54] reported an analysis of the effects of JA on tobacco BY-2 cells, finding that transcript levels were increased for 12 ethylene response factors and 4 basic helix-loop-helix factors associated with alkaloid formation. JAs are known to operate in complex networks with crosstalk to other phytohormone signaling pathways in the regulation of tobacco nicotine biosynthesis. Examples include the JA ZIM-domain 1(JAZ1) protein (a key repressor of JA signaling) which interacts in vivo with DELLA proteins (repressors of the GA pathway) [48], and *NtPYL4* (a functional ABA receptor) whose transcription is regulated by JAs [49].

In the present study, we propose that topping, as a form of mechanical wounding, activates JA signaling as one of the earliest events. JA-Ile, as the only JA derivative known to be involved in JA signaling, displayed continuously increasing levels over the first 24 h after topping, indicating a vital role in the regulation of nicotine biosynthesis. The expression levels of the two JAZ-encoding genes were significantly up-regulated at t = 0.5 h and t = 8 h, respectively, which is consistent with the observed increases in JA and JA-Ile levels. Our experimental data also indicated similar patterns of changes in concentrations of JA and IAA following topping. This is consistent with the report that JA and auxin have very similar signal transmission mechanisms. However, how JA and IAA collaboratively respond to topping stimulation and regulate nicotine biosynthesis remains unclear [37], and will require further investigation.

As a consequence of phytohormone changes, genes in the downstream of regulation network also showed corresponding variation. Notably, all the genes encoding AUX/IAA were found to be up-regulated at t = 0.5 h, which implies that IAA levels increased at an early time point. Similarly, all ABA receptor PYL genes exhibited lower expression levels at t = 0.5 h and t = 8 h, suggesting that altered *PYL* expression affects the JA response to nicotine biosynthesis in tobacco. Finally, in our study, we found that a large number of regulatory genes associated with auxin signaling, ABA signaling, ethylene signaling, and gibberellin signaling showed significantly altered expression levels at t = 0.5 h after topping, indicating the complex networks with crosstalk are promptly constructed across these phytohormonal signaling pathways as an early response to topping (Additional file 11: Table S6).

## Conclusions

By sequencing the transcriptomes of *N. tabacum* roots at different points following topping, we identified 4830 genes showing differential expression levels, including 11 gene families involved in nicotine biosynthesis and transport, 549 genes encoding transcriptional factors, and 336 genes in phytohormone signal transduction. This provides excellent candidates for future functional genomics studies to illustrate the biosynthesis and regulation of nicotine in tobacco roots.

## Methods

### RNA extraction, library preparation, and transcriptome sequencing

The seeds of *Nicotiana tabacum* Yunyan 87 were kindly provided by the Tobacco Seed Bank of the Yunnan Academy of Tobacco Agricultural Sciences. The plants were identified by Dr. Zhongbang Song. The voucher specimen is accessible at the Herbarium of the Kunming Institute of Botany, Chinese Academy of Sciences (accessions No. SGL-001-1). All the materials were grown for 8 weeks in commercial potting soil in a Percival PGC-10 incubator set for a 16 h day/8 h night cycle at 28 °C. Individual plants with the most similar morphology were selected for topping by removing the apices above the youngest unfolded leaf. Roots were washed with water, and tissues excised using a surgical blade, 18 samples being taken 0, 0.5, 1, 3, 5, 8 and 24 h after topping, with 2–3 replicates at each time point (Additional file 6: Table S1). For RNA extraction, root tissues were frozen in liquid nitrogen and ground into a powder using a mortar and pestle. RNA was extracted using TRIzol (Invitrogen Life Technologies) according to the manufacturer’s instructions. Extracted RNA was assessed for quality and quantity using an Agilent 2100 Bioanalyzer (Agilent Technologies) and were proceeded for library construction. The amplified libraries were sequenced on an Illumina HiSeq™ 2000 sequencing machine in October 2013 at BGI (Shenzhen, China). Reads were generated in 90 bp paired-end format. The sequencing data was deposited in the National Center for Biotechnology Information (NCBI) Sequence Read Archive (SRA) database with accession number SRP154415.

### Mapping of sequencing reads and quantification of gene expression

Before further analysis, the raw reads were filtered to remove adaptor sequences, low-quality reads, and reads containing poly-N, using CutAdapt (http://code.google.com/p/cutadapt/) and Btrim [55]. Next, we processed and mapped the clean reads onto the *Nicotiana tabacum* genome [56] using Hisat2 version 2.1.0 [57] with default parameters. The reference genome can be found on the Sol Genomics Network website (ftp://ftp.solgenomics.net/genomes/Nicotiana_tabacum/edwards_et_al_2017/assembly/Nitab-v4.5_genome_Chr_Edwards2017.fasta.gz). Gene expression levels were estimated as FPKM (fragments per kilobase of exon per million fragments mapped reads) values [58] using Cufflinks software version 1.2.1 [59].

### Identification and enrichment analysis of DEGs

Differential expression of genes between the t = 0 h sample and other samples at different time points were calculated with the cuffdiff program in Cufflinks [59]. Gene abundance differences among these samples were established on basis of the fold change (FC) of the FPKM values. The genes with an absolute value of log_2_ fold changes (FC) ≥ 1 and an adjusted *p*-value (q-value) ≤ 0.05 were defined as differentially expressed genes (DEGs). The FPKM values of the DEGs were normalized and clustered by pheatmap with parameters “scale=row, cluster_rows=T, cutree_rows=8”, where cutree function in R was used to estimate category number and eight categories were obtained when h = 4.8.

Gene Ontology (GO) and Kyoto Encyclopedia of Genes and Genomes (KEGG) analyses were performed to identify the enrichment of DEGs in GO terms and metabolic pathways, respectively. For functional categorization and pathway visualization of DEGs, the metabolic pathways of DEGs were predicted using the KEGG Automated Annotation Server (KAAS) with default parameters. The DEGs in the KEGG pathways and GO analysis were enriched using ClusterProfile R and WEGO [60, 61]. A corrected *p*-value (*q*-value) ≤ 0.05 was chosen as the threshold for significantly enriched KEGG pathways and GO terms.

### Clustering of differentially expressed TFs and the DEGs involved in nicotine biosynthesis by expression profiles

Clustering of differentially expressed TFs and the DEGs involved in nicotine biosynthesis was performed using R package mfuzz with the cluster number assigned to 15 using the Dmin function and others as defaults on the profiles of FPKM at the different time points after topping.

### Subgenome assignment of all the DEGs

All DEGs were used as queries to search against all the annotated proteins in the genomes of *N. tomentosiformis* and *N. sylvestris*. Subgenomes were assigned based on the first hit species if the first hit had at least 90% identity and the second hit showed a lower identity value than the first hit. For the DEGs involved in nicotine biosynthesis and transport, a manual check was performed based on previous reports.

### Quantitative real-time PCR analysis (qRT-PCR)

To confirm the gene expression levels of the RNA-Seq assay, 1 μg total RNA mixed equally from 3 plants for each RNA-Seq sample was used for the qRT-PCR assay as previously described [62]. The RNA, following elimination of residual genomic DNA using DNase I, was reverse transcribed into cDNA using the PrimeScript RT reagent Kit with gDNA Eraser (Perfect Real Time) (Takara, Japan). qRT-PCR was performed in a 20 μL reaction volume using the LC480 system (Roche, SUI) and with three biological replicates. Three technical replicates were used for the samples at different time points with the tobacco *Actin* gene as an internal standard for normalization, and SYBR Green as the fluorochrome. A three-step PCR process was performed with a pre-denaturation at 95 °C for 30 s, followed by 45 cycles of denaturation at 95 °C for 30 s, annealing at the optimal temperature of each primer pair for 20 s, and elongation at 72 °C for 20 s, and finally for melting point curve analysis (95 °C for 15 s, 60 °C for 1 min and 95 °C for 15 s) to test amplicon specificity. Relative quantification of gene expression level was carried out using the 2^-△△Ct^ method. The specific primer sequences of the selected genes for qRT-PCR validation were designed based on the divergent regions among the orthologous genes and listed in Additional file 8: Table S7.

### Measurement of nicotine content and hormone levels

For measurement of nicotine content, roots were dried at 105^o^ C for 30 min and at 60^o^ C for 3d. Each sample (100 mg) equally mixed from 4 individual plants was mixed with 1 mL of extraction solution (40% methanol containing 0.5% acetic acid (v/v)), and nicotine was quantified by HPLC as previously described [63]. Three biological replicates each with four technical replicates were performed. For quantification of phytohormones, approximately 200 mg of tobacco roots were ground in liquid nitrogen and 1 mL of ethyl acetate spiked with the internal standards was added to each sample. ^13^C_2_-JA, ^13^C_2_-JA-Ile, D_4_-SA, D_6_-ABA and D_5_-IAA were used as the internal standards for JA, JA-Ile, SA, ABA and IAA, respectively. Phytohormone extraction and quantification were described as previously by HPLC-MS/MS (LCMS-8040, Shimadzu) system [64]. Three biological replicates were used for each sample.

## Supplementary information

**Additional file 1: Figure S1.** The FPKM density of each sample after topping in tobacco. The horizontal axis indicates the sample log_10_(FPKM) and the vertical axis indicates the corresponding probability density. The different colors denote different samples.

**Additional file 2: Figure S2.** Correlation of different biological replicates across all samples.

**Additional file 3: Figure S3.** Hierarchical cluster analysis of 4830 DEGs from different time points after topping in tobacco. The samples and treatments are indicated below each column. DEGs are defined by different colors, with the normalized expression levels employing a color gradient from low (blue) to high (red).

**Additional file 4: Figure S4.** Classification statistics of DEGs according to the GO annotations. The GO classification is indicated on the abscissa, with the ordinate providing the percentage of the number of genes (left) and the number of genes (right).

**Additional file 5: Figure S5.** KEGG overview of DEGs after topping in tobacco. The numbers in the picture represent the DEGs that were annotated to the metabolic pathway, with the percentage representing the ratio of this count to the number of DEGs annotated to the total metabolic pathway.

**Additional file 6: Table S1.** Quality and mapping rates of RNA-Seq data.

**Additional file 7: Table S2.** List of DEGs in roots after topping.

**Additional file 8: Table S3.** DEGs involved in nicotine biosynthesis and transport after topping.

**Additional file 9: Table S4.** Differentially transcribed transcription factors after topping.

**Additional file 10: Table S5.** Clustering TFs and up-regulated gene families in nicotine biosynthesis according to expression pattern after topping.

**Additional file 11: Table S6.** DEGs involved in phytohormone signal transduction after topping.

**Additional file 12: Table S7.** Sequence-specific primers used in qRT-PCR.

## Data Availability

The sequencing data were then deposited in the National Center for Biotechnology Information (NCBI) Sequence Read Archive (SRA) database with the accession number SRP154415.

## Abbreviations

A622: Isoflavone reductase-like protein
ABA: Abscisic acid
ADC: Arginine decarboxylase
AO: Aspartate oxidase
AP2-EREBP: Ethylene responsive transcription factor
ARF: Auxin response factor
BBL: Berberine bridge enzyme-like protein
CDPK: Calcium-dependent protein kinase
CIPK: CBL-interacting protein kinase
DEGs: Differentially-expressed genes
ETR: Ethylene receptor
FPKM: Fragments Per Kilobase of transcript per Million fragments mapped
GO: Gene Ontology
IAA: Auxin
JA: Jasmonic acid
JAZ1: JA ZIM-domain 1 containing protein, jasmonoyl isoleucine (JA-Ile).
KEGG: Kyoto Encyclopedia of Genes and Genomes
MPO: N-methylputrescine oxidase
NAD: Nicotinic acid dinucleotide
ODC: Ornithine decarboxylase
PMT: Putrescine N-methyltransferase
PP2C: Protein phosphatase 2C
QPT: Quinolinic acid phosphoribosyl transferase
QS: Quinolinate synthase
SAMDC: S-adenosylmethionine decarboxylase
SAMS: S-adenosyl-L-methionine synthetase
SSH: Suppression subtractive hybridization
TF: Transcription factors
VIGS: Virus induced gene silencing

## References

[1] Baldwin IT (2001). An ecologically motivated analysis of plant-herbivore interactions in native tobacco. Plant Physiol.

[2] Baldwin IT, Karb MJ, Ohnmeiss TE (1994). Allocation of N-15 from nitrate to nicotine - production and turnover of a damage-induced Mobile defense. Ecol.

[3] Baldwin IT, Schmelz EA, Ohnmeiss TE (1994). Wound-induced changes in root and shoot Jasmonic acid pools correlate with induced nicotine synthesis in *Nicotiana-Sylvestris Spegazzini* and comes. J Chem Ecol.

[4] Kutchan TM (1995). Alkaloid biosynthesis - the basis for metabolic engineering of medicinal plants. Plant Cell.

[5] Chen X, Sun S, Liu F, Shen E, Liu L, Ye C (2019). A transcriptomic profile of topping responsive non-coding RNAs in tobacco roots (*Nicotiana tabacum*). BMC Genomics.

[6] Wang SS, Shi QM, Li WQ, Niu JF, Li CJ, Zhang FS (2008). Nicotine concentration in leaves of flue-cured tobacco plants as affected by removal of the shoot apex and lateral buds. J Integr Plant Biol.

[7] Hakkinen ST, Tilleman S, Swiatek A, De Sutter V, Rischer H, Vanhoutte I, Van Onckelen H, Hilson P, Inze D, Oksman-Caldentey K-M (2007). Functional characterisation of genes involved in pyridine alkaloid biosynthesis in tobacco. Phytochemistry.

[8] Tiburcio AF, Galston AW (1986). Arginine decarboxylase as the source of putrescine for tobacco alkaloids. Phytochemistry.

[9] Imanishi S, Hashizume K, Nakakita M, Kojima H, Matsubayashi Y, Hashimoto T, Sakagami Y, Yamada Y, Nakamura K (1998). Differential induction by methyl jasmonate of genes encoding ornithine decarboxylase and other enzymes involved in nicotine biosynthesis in tobacco cell cultures. Plant Mol Biol.

[10] Saunders JW, Bush LP (1979). Nicotine biosynthetic enzyme activities in *Nicotiana tabacum L.* genotypes with different alkaloid levels. Plant Physiol.

[11] Hibi N, Higashiguchi S, Hashimoto T, Yamada Y (1994). Gene expression in tobacco low-nicotine mutants. Plant Cell.

[12] Katoh A, Shoji T, Hashimoto T (2007). Molecular cloning of N-methylputrescine oxidase from tobacco. Plant Cell Physiol.

[13] Heim WG, Sykes KA, Hildreth SB, Sun J, Lu RH, Jelesko JG (2007). Cloning and characterization of a *Nicotiana tabacum* methylputrescine oxidase transcript. Phytochemistry.

[14] Wagner R, Feth F, Wagner KG (1986). Regulation in tobacco callus of enzyme activities of the nicotine pathway : II. The pyridine-nucleotide cycle. Planta.

[15] Sinclair SJ, Murphy KJ, Birch CD, Hamill JD (2000). Molecular characterization of quinolinate phosphoribosyltransferase (QPRTase) in Nicotiana. Plant Mol Biol.

[16] Katoh A, Uenohara K, Akita M, Hashimoto T (2006). Early steps in the biosynthesis of NAD in Arabidopsis start with aspartate and occur in the plastid. Plant Physiol.

[17] Kajikawa M, Shoji T, Kato A, Hashimoto T (2011). Vacuole-localized Berberine bridge enzyme-like proteins are required for a late step of nicotine biosynthesis in tobacco. Plant Physiol.

[18] DeBoer KD, Lye JC, Aitken CD, Su AKK, Hamill JD (2009). The A622 gene in *Nicotiana glauca* (tree tobacco): evidence for a functional role in pyridine alkaloid synthesis. Plant Mol Biol.

[19] Qi YC, Guo HX, Li K, Liu WQ (2012). Comprehensive analysis of differential genes and miRNA profiles for discovery of topping-responsive genes in flue-cured tobacco roots. FEBS J.

[20] Xu S, Brockmoller T, Navarro-Quezada A, Kuhl H, Gase K, Ling Z, Zhou W, Kreitzer C, Stanke M, Tang H (2017). Wild tobacco genomes reveal the evolution of nicotine biosynthesis. Proc Natl Acad Sci U S A.

[21] Kajikawa M, Sierro N, Kawaguchi H, Bakaher N, Ivanov NV, Hashimoto T, Shoji T (2017). Genomic insights into the evolution of the nicotine biosynthesis pathway in tobacco. Plant Physiol.

[22] Todd AT, Liu EW, Polvi SL, Pammett RT, Page JE (2010). A functional genomics screen identifies diverse transcription factors that regulate alkaloid biosynthesis in *Nicotiana benthamiana*. Plant J.

[23] Han S, Wang CW, Wang WL, Jiang J (2014). Mitogen-activated protein kinase 6 controls root growth in Arabidopsis by modulating Ca^2+^ −based Na^+^ flux in root cell under salt stress. J Plant Physiol.

[24] Li K, Yang F, Zhang G, Song S, Li Y, Ren D, Miao Y, Song CP (2017). AIK1, a mitogen-activated protein kinase, modulates Abscisic acid responses through the MKK5-MPK6 kinase Cascade. Plant Physiol.

[25] Li K, Yang F, Miao Y, Song CP (2017). Abscisic acid signaling is involved in regulating the mitogen-activated protein kinase cascade module, AIK1-MKK5-MPK6. Plant Signal Behav.

[26] Chintapakorn Y, Hamill JD (2003). Antisense-mediated down-regulation of putrescine N-methyltransferase activity in transgenic *Nicotiana tabacum L*. can lead to elevated levels of anatabine at the expense of nicotine. Plant Mol Biol.

[27] Fu Y, Guo H, Cheng Z, Wang R, Li G, Huo G, Liu W (2013). NtNAC-R1, a novel NAC transcription factor gene in tobacco roots, responds to mechanical damage of shoot meristem. Plant Physiol Biochem.

[28] Dalton HL, Blomstedt CK, Neale AD, Gleadow R, DeBoer KD, Hamill JD (2016). Effects of down-regulating ornithine decarboxylase upon putrescine-associated metabolism and growth in *Nicotiana tabacum L*. J Exp Bot.

[29] DeBoer KD, Dalton HL, Edward FJ, Hamill JD (2011). RNAi-mediated down-regulation of ornithine decarboxylase (ODC) leads to reduced nicotine and increased anatabine levels in transgenic *Nicotiana tabacum L*. Phytochemistry.

[30] Xu BF, Timko MP (2004). Methyl jasmonate induced expression of the tobacco putrescine N-methyltransferase genes requires both G-box and GCC-motif elements. Plant Mol Biol.

[31] Riechers DE, Timko MP (1999). Structure and expression of the gene family encoding putrescine N-methyltransferase in *Nicotiana tabacum*: new clues to the evolutionary origin of cultivated tobacco. Plant Mol Biol.

[32] Shoji T, Nakajima K, Hashimoto T (2000). Ethylene suppresses Jasmonate-induced gene expression in nicotine biosynthesis. Plant Cell Physiol.

[33] Ryan SM, Cane KA, DeBoer KD, Sinclair SJ, Brimblecombe R, Hamill JD (2012). Structure and expression of the quinolinate phosphoribosyltransferase (QPT) gene family in Nicotiana. Plant Sci.

[34] Shoji T, Hashimoto T (2011). Recruitment of a duplicated primary metabolism gene into the nicotine biosynthesis regulon in tobacco. Plant J.

[35] Kajikawa M, Hirai N, Hashimoto T (2009). A PIP-family protein is required for biosynthesis of tobacco alkaloids. Plant Mol Biol.

[36] Sierro N, Battey JND, Ouadi S, Bovet L, Goepfert S, Bakaher N, Peitsch MC, Ivanov NV. Reference genomes and transcriptomes of *Nicotiana sylvestris* and *Nicotiana tomentosiformis*. Genome Biol. 2013;14(6). 10.1186/gb-2013-14-6-r60 PMC3707018 23773524

[37] Jin WH, Zhou Q, Wei YF, Yang JM, Hao FS, Cheng ZP, Guo HX, Liu WQ. NtWRKY-R1, a novel transcription factor, integrates IAA and JA signal pathway under topping damage stress in *Nicotiana tabacum*. Front Plant Sci. 2018;8. 10.3389/fpls.2017.02263 PMC5775218 29379516

[38] Wang XW, Bennetzen JL (2015). Current status and prospects for the study of Nicotiana genomics, genetics, and nicotine biosynthesis genes. Mol Gen Genomics.

[39] Rushton PJ, Bokowiec MT, Han S, Zhang H, Brannock JF, Chen X, Laudeman TW, Timko MP (2008). Tobacco transcription factors: novel insights into transcriptional regulation in the Solanaceae. Plant Physiol.

[40] Sears MT, Zhang HB, Rushton PJ, Wu M, Han SC, Spano AJ, Timko MP (2014). NtERF32: a non-NIC2 locus AP2/ERF transcription factor required in jasmonate-inducible nicotine biosynthesis in tobacco. Plant Mol Biol.

[41] Goossens J, Fernandez-Calvo P, Schweizer F, Goossens A (2016). Jasmonates: signal transduction components and their roles in environmental stress responses. Plant Mol Biol.

[42] Zhang HB, Bokowiec MT, Rushton PJ, Han SC, Timko MP (2012). Tobacco transcription factors NtMYC2a and NtMYC2b form nuclear complexes with the NtJAZ1 repressor and regulate multiple Jasmonate-inducible steps in nicotine biosynthesis. Mol Plant.

[43] Shoji T, Hashimoto T (2011). Tobacco MYC2 regulates Jasmonate-inducible nicotine biosynthesis genes directly and by way of the NIC2-locus ERF genes. Plant Cell Physiol.

[44] Wang B, Lewis RS, Shi J, Song Z, Gao Y, Li W, Chen H, Qu R (2015). Genetic factors for enhancement of nicotine levels in cultivated tobacco. Sci Rep.

[45] Han Y, Wu M, Cao L, Yuan W, Dong M, Wang X, Chen W, Shang F (2016). Characterization of OfWRKY3, a transcription factor that positively regulates the carotenoid cleavage dioxygenase gene OfCCD4 in *Osmanthus fragrans*. Plant Mol Biol.

[46] Wang P, Yang C, Chen H, Luo L, Leng Q, Li S, Han Z, Li X, Song C, Zhang X, Wang D (2018). Exploring transcription factors reveals crucial members and regulatory networks involved in different abiotic stresses in *Brassica napus* L. BMC Plant Biol.

[47] Xiao L, Carrillo J, Siemann E, Ding J (2019). Herbivore-specific induction of indirect and direct defensive responses in leaves and roots. AoB PLANTS.

[48] Hou XL, Lee LYC, Xia KF, Yen YY, Yu H (2010). DELLAs modulate Jasmonate signaling via competitive binding to JAZs. Dev Cell.

[49] Lackman P, Gonzalez-Guzman M, Tilleman S, Carqueijeiro I, Perez AC, Moses T, Seo M, Kanno Y, Hakkinen ST, Van Montagu MCE (2011). Jasmonate signaling involves the abscisic acid receptor PYL4 to regulate metabolic reprogramming in Arabidopsis and tobacco. P Natl Acad Sci USA.

[50] De Boer K, Tilleman S, Pauwels L, Vanden Bossche R, De Sutter V, Vanderhaeghen R, Hilson P, Hamill JD, Goossens A (2011). APETALA2/ETHYLENE RESPONSE FACTOR and basic helix-loop-helix tobacco transcription factors cooperatively mediate jasmonate-elicited nicotine biosynthesis. Plant J.

[51] Palazon J, Pinol MT, Altabella T, Cusido R, Serrano M (1987). Auxin-induced regulation of amino-acid and Putrescine in the Free State and nicotine content in cultured tobacco callus. J Plant Physiol.

[52] Xu BF, Sheehan MJ, Timko MP (2004). Differential induction of ornithine decarboxylase (ODC) gene family members in transgenic tobacco (*Nicotiana tabacum L*. cv. *Bright Yellow 2*) cell suspensions by methyl-jasmonate treatment. Plant Growth Regul.

[53] Shoji T, Yamada Y, Hashimoto T (2000). Jasmonate induction of Putrescine N-methyltransferase genes in the root of *Nicotiana sylvestris*. Plant Cell Physiol.

[54] Yang Y, Yan P, Yi C, Li W, Chai Y, Fei L, Gao P, Zhao H, Wang Y, Timko MP (2017). Transcriptome-wide analysis of jasmonate-treated BY-2 cells reveals new transcriptional regulators associated with alkaloid formation in tobacco. J Plant Physiol.

[55] Kong Y (2011). Btrim: a fast, lightweight adapter and quality trimming program for next-generation sequencing technologies. Genomics.

[56] Edwards KD, Fernandez-Pozo N, Drake-Stowe K, Humphry M, Evans AD, Bombarely A, Allen F, Hurst R, White B, Kernodle SP (2017). A reference genome for *Nicotiana tabacum* enables map-based cloning of homeologous loci implicated in nitrogen utilization efficiency. BMC Genomics.

[57] Kim D, Langmead B, Salzberg SL (2015). HISAT: a fast spliced aligner with low memory requirements. Nat Methods.

[58] Mortazavi A, Williams BA, McCue K, Schaeffer L, Wold B (2008). Mapping and quantifying mammalian transcriptomes by RNA-Seq. Nat Methods.

[59] Roberts A, Pimentel H, Trapnell C, Pachter LJB (2011). Identification of novel transcripts in annotated genomes using RNA-Seq. Bioinformatics.

[60] Ye J, Zhang Y, Cui H, Liu J, Wu Y, Cheng Y (2018). WEGO 2.0: a web tool for analyzing and plotting GO annotations. Nucleic Acids Res.

[61] Yu G, Wang LG, Han Y, He QY (2012). ClusterProfiler: an R package for comparing biological themes among gene clusters. Omics.

[62] Qiu DY, Bai SL, Ma JC, Zhang LS, Shao FJ, Zhang KK, Yang YF, Sun T, Huang JL, Zhou Y (2019). The genome of *Populus alba x Populus tremulavar. glandulosa* clone 84K. DNA Res.

[63] Onkokesung N, Gaquerel E, Kotkar H, Kaur H, Baldwin IT, Galis I (2012). MYB8 controls inducible phenolamide levels by activating three novel hydroxycinnamoyl-coenzyme a:polyamine transferases in *Nicotiana attenuata*. Plant Physiol.

[64] Wu J, Hettenhausen C, Meldau S, Baldwin IT (2007). Herbivory rapidly activates MAPK signaling in attacked and unattacked leaf regions but not between leaves of *Nicotiana attenuata*. Plant Cell.

