# Supplementary figures and images for: Transcriptome analysis reveals key genes involved in the regulation of nicotine biosynthesis at early time points after topping in tobacco (Nicotiana tabacum L.)
Source: BMC Plant Biol. 2020 Jan 20;20:30. doi: 10.1186/s12870-020-2241-9 (PMC6971868; doi:10.1186/s12870-020-2241-9)

genes

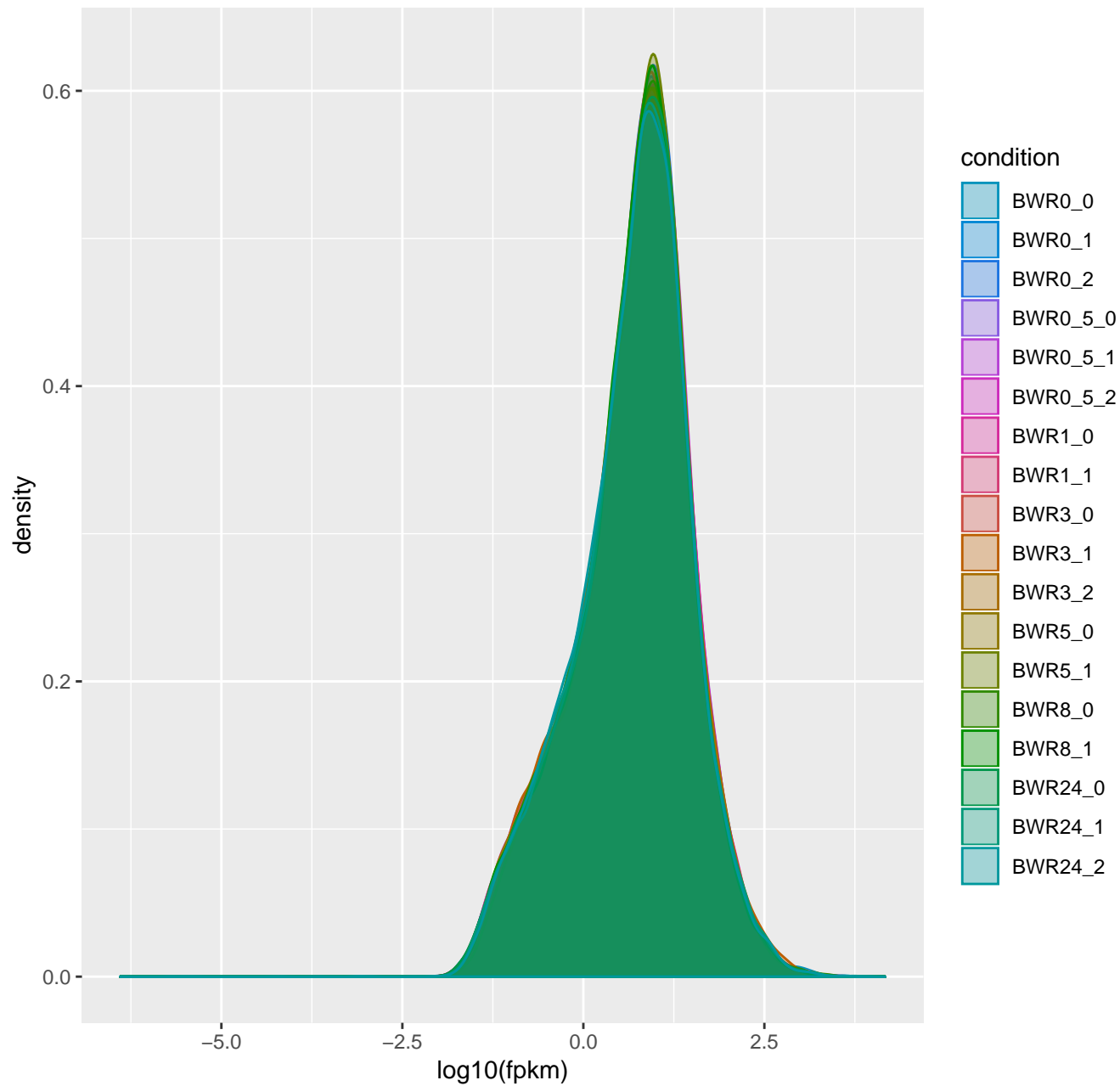

Supplement: Supplementary file 1 — Additional file 1: Figure S1. The FPKM density of each sample after topping in tobacco. The horizontal axis indicates the sample log10(FPKM) and the vertical axis indicates the corresponding probability density. The different colors denote different samples. [file 12870_2020_2241_MOESM1_ESM.pdf]

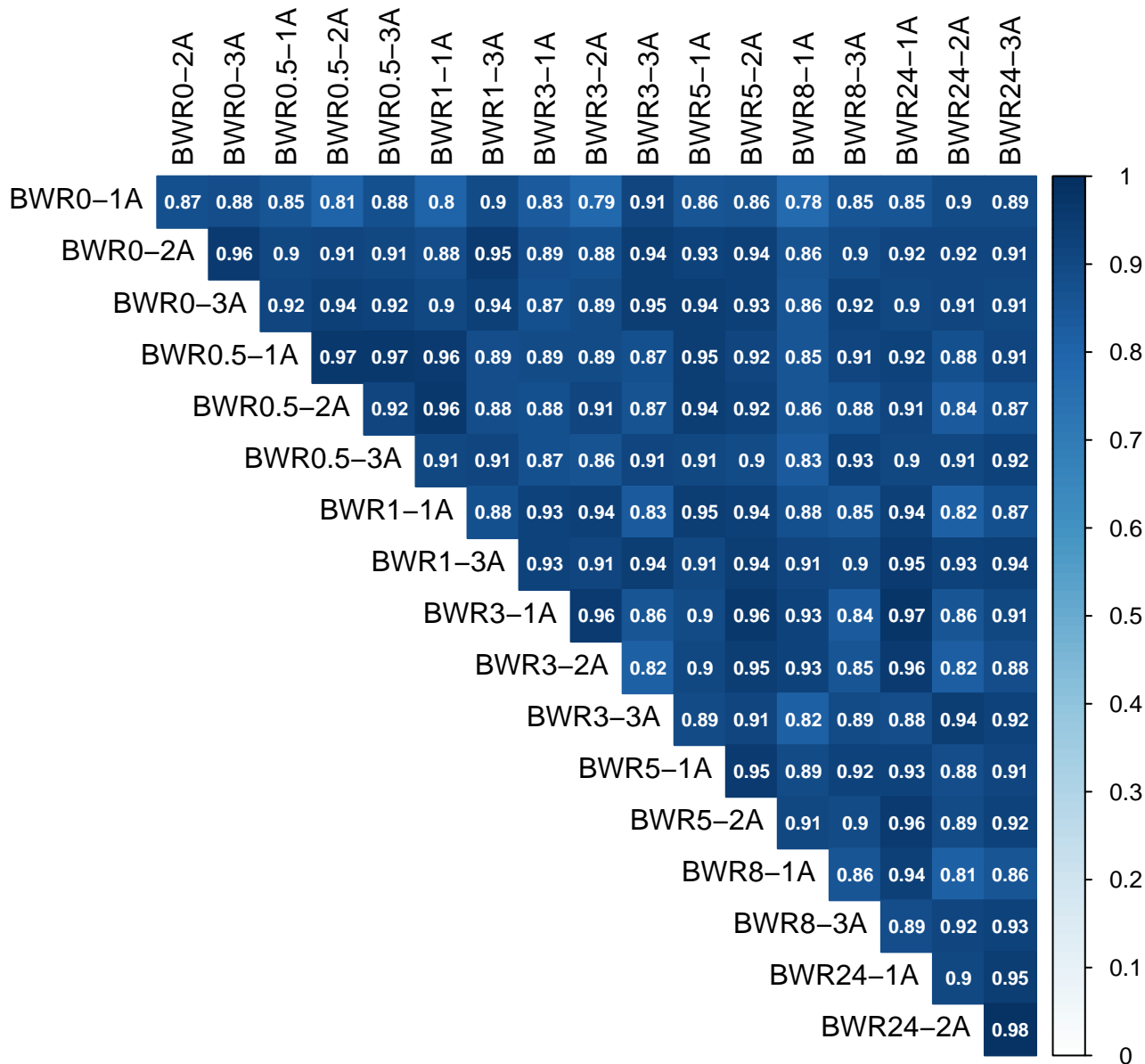

Supplement: Supplementary file 2 — Additional file 2: Figure S2. Correlation of different biological replicates across all samples. [file 12870_2020_2241_MOESM2_ESM.pdf]

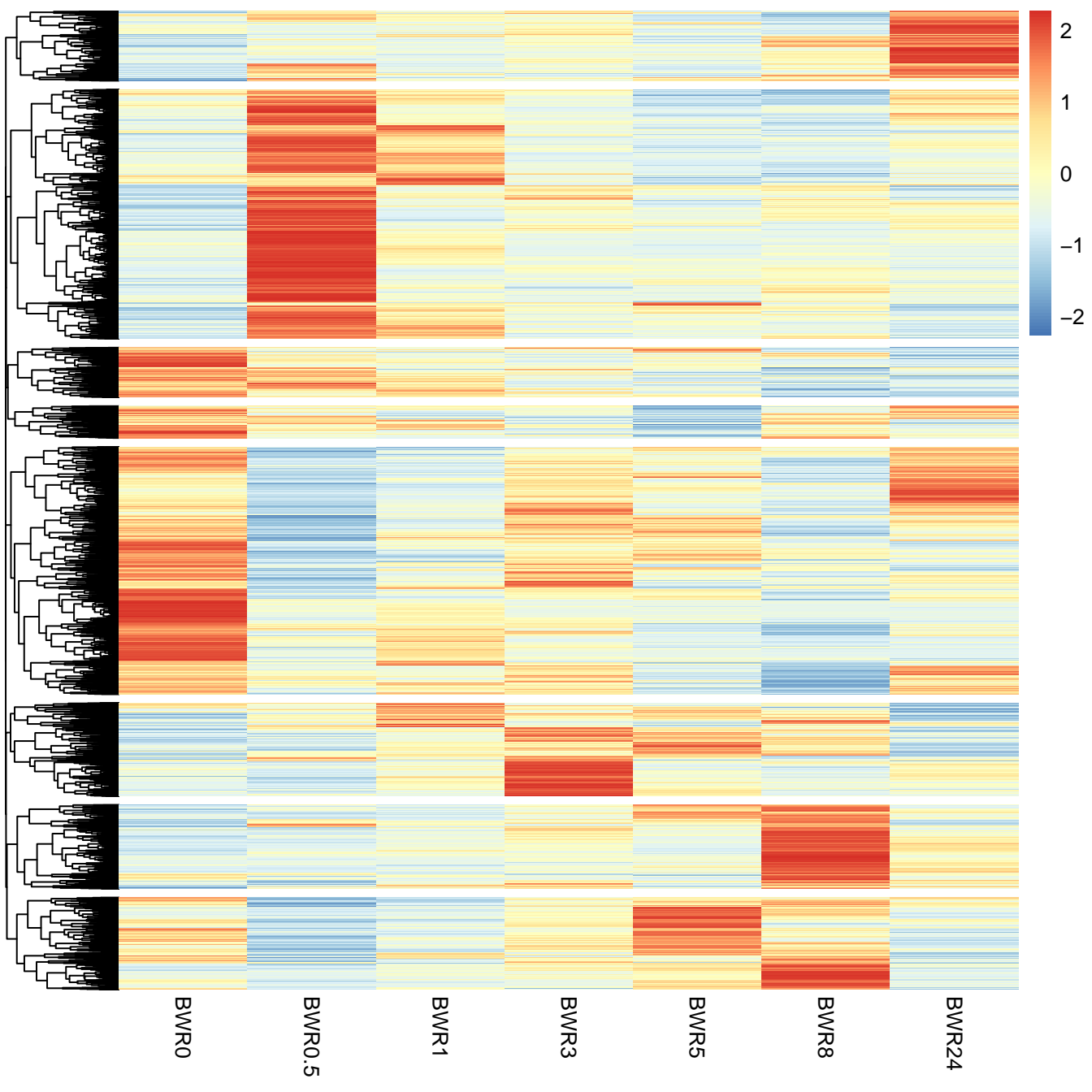

Supplement: Supplementary file 3 — Additional file 3: Figure S3. Hierarchical cluster analysis of 4830 DEGs from different time points after topping in tobacco. The samples and treatments are indicated below each column. DEGs are defined by different colors, with the normalized expression levels employing a color gradient from low (blue) to high (red). [file 12870_2020_2241_MOESM3_ESM.pdf]

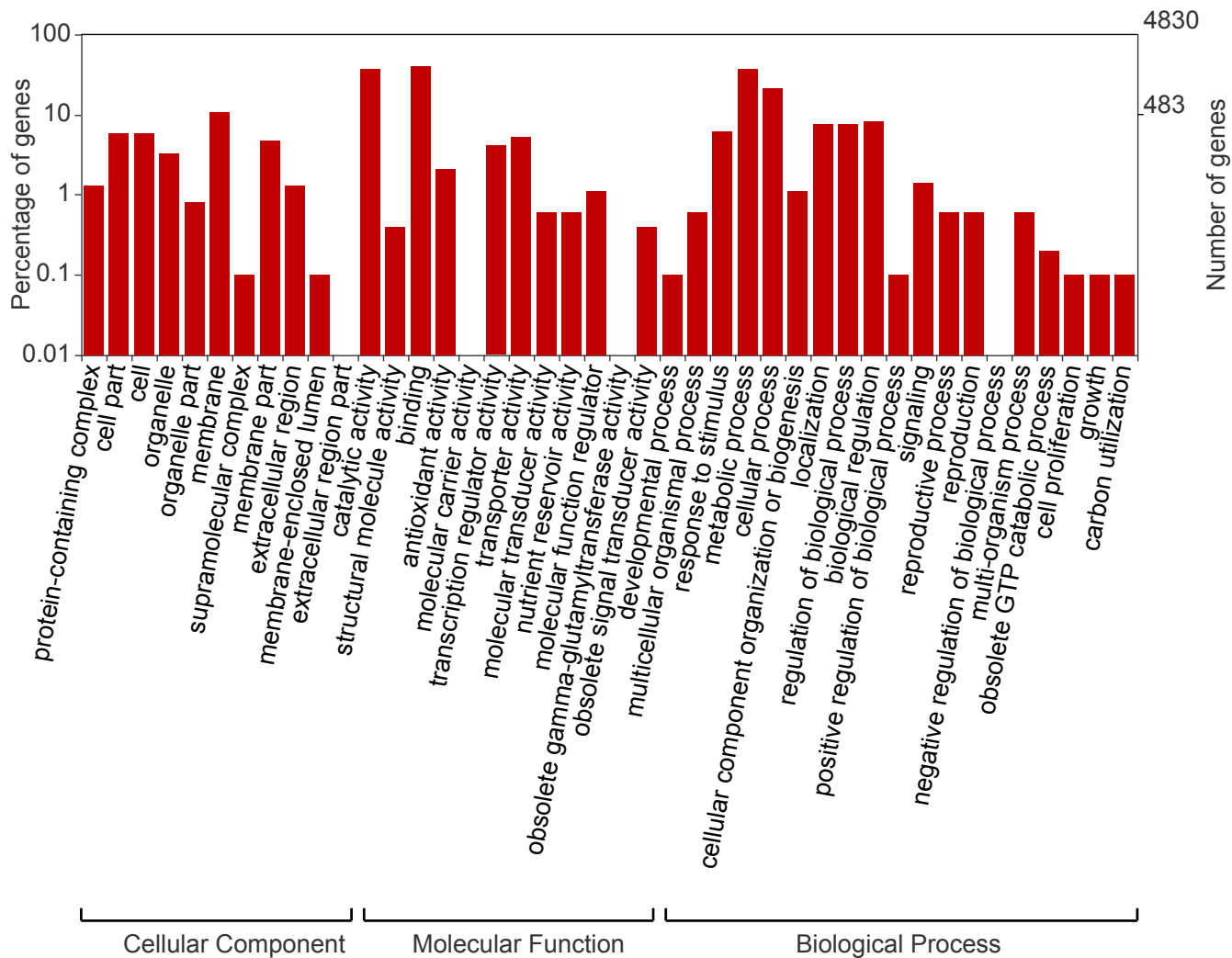

Supplement: Supplementary file 4 — Additional file 4: Figure S4. Classification statistics of DEGs according to the GO annotations. The GO classification is indicated on the abscissa, with the ordinate providing the percentage of the number of genes (left) and the number of genes (right). [file 12870_2020_2241_MOESM4_ESM.pdf]

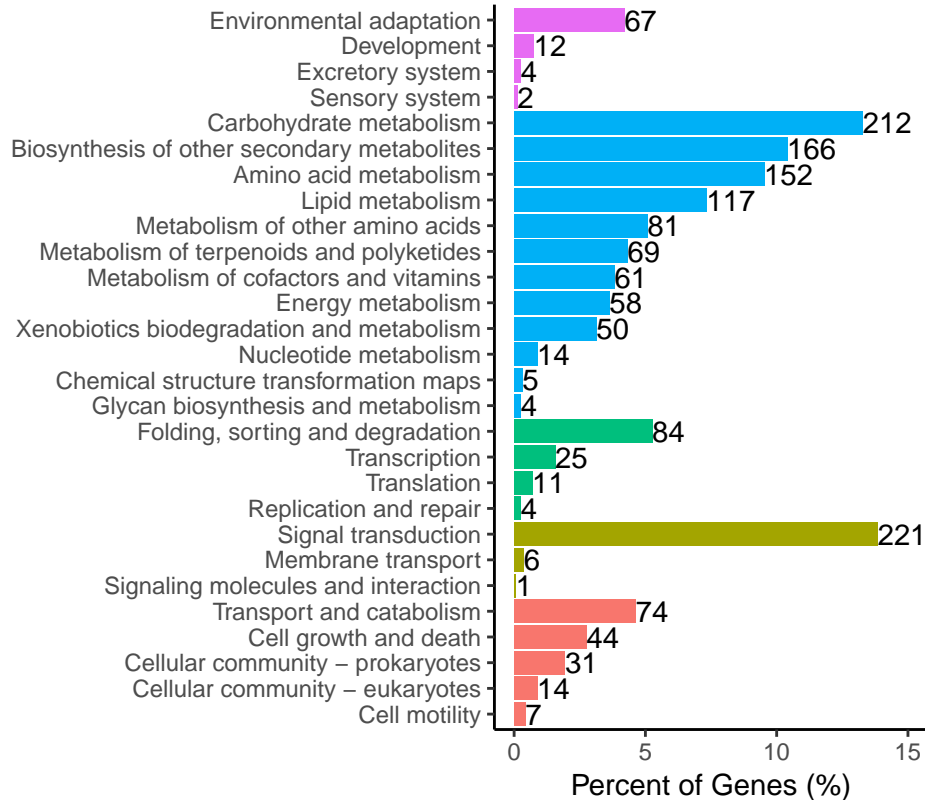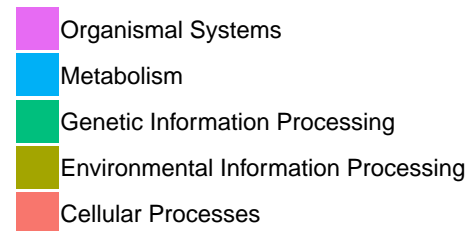

Supplement: Supplementary file 5 — Additional file 5: Figure S5. KEGG overview of DEGs after topping in tobacco. The numbers in the picture represent the DEGs that were annotated to the metabolic pathway, with the percentage representing the ratio of this count to the number of DEGs annotated to the total metabolic pathway. [file 12870_2020_2241_MOESM5_ESM.pdf]
