# Supplementary material for: Transcriptome analysis reveals key genes involved in the regulation of nicotine biosynthesis at early time points after topping in tobacco (Nicotiana tabacum L.)
Source: BMC Plant Biol. 2020 Jan 20;20:30. doi: 10.1186/s12870-020-2241-9 (PMC6971868; doi:10.1186/s12870-020-2241-9)
Supplement: Supplementary file 12 — Additional file 12: Table S7. Sequence-specific primers used in qRT-PCR. [file 12870_2020_2241_MOESM12_ESM.docx]

**Table S6.** Sequence-specific primers used for qRT-PCR

| **Gene ID** | **Annotation** | **Primer-F (5'-3')** | **Primer-R (5'-3')** |
| --- | --- | --- | --- |
| Nitab4.5_0004607g0020 | *Actin* | CTGAGGTCCTTTTCCAACCA | TACCCGGGAACATGGTAGAG |
| Nitab4.5_0000013g0390 | *PMT1-1* | CCGGTGCGGAGTCAAATTAT | TGGACTTGACTTGAGTTGTCTCT |
| Nitab4.5_0000013g0380 | *PMT1-2* | AATGGCACTTCCGAACATCG | GTTGCCATTGTCATGGCTGA |
| Nitab4.5_0000093g0170 | *bHLH25* | TAGCCAAGCCACGAAATTGG | AGACCCGGAAGAATTGCTGA |
| Nitab4.5_0003280g0010 | *ODC-1* | GTGCTAGACTTGGGTGAGGT | GAAAGGAACGACGGTTCAGG |
| Nitab4.5_0003721g0020 | *ETR2* | GGCTAATCAGGTTGCTGTGG | GCATCCTGCTTTGCTTGTT |
| Nitab4.5_0000025g0290 | *MPO* | agtcgtttcaactcctcccgtaa | agaagatggccttggaaagaatg |
| Nitab4.5_0005632g0010 | *PP2C* | gtcacaccaacggtctcaggggat | ctctcttcaaatcaacgacgacaac |
| Nitab4.5_0026691g0010 | *PYL4* | Tgccgacatcaccgtcccctac | tatggtcagtggtccgccgcttc |
| Nitab4.5_0004657g0030 | *ARF7* | GAAGACACAACGGCAACACT | GGACGATCTTCAGCAGAGGT |
